# Colon Cancer and Perturbations of the Sphingolipid Metabolism

**DOI:** 10.3390/ijms20236051

**Published:** 2019-11-30

**Authors:** Miroslav Machala, Jiřina Procházková, Jiřina Hofmanová, Lucie Králiková, Josef Slavík, Zuzana Tylichová, Petra Ovesná, Alois Kozubík, Jan Vondráček

**Affiliations:** 1 Department of Chemistry and Toxicology, Veterinary Research Institute, Hudcova 296/70, 62100 Brno, Czech Republic; prochazkova.j@vri.cz (J.P.); kralikova@vri.cz (L.K.); slavik@vri.cz (J.S.); 2 Department of Cytokinetics, Institute of Biophysics of the Czech Academy of Sciences, Královopolská 135, 61265 Brno, Czech Republic; hofmanova@ibp.cz (J.H.); tylichova@ibp.cz (Z.T.); ovesna@iba.muni.cz (P.O.); kozubik@ibp.cz (A.K.); vondracek@ibp.cz (J.V.); 3 Institute of Biostatistics and Analyses, Faculty of Medicine, Masaryk University, Poštovská 68/3, 60200 Brno, Czech Republic

**Keywords:** colorectal cancer, colon cancer cells, sphingolipid, glycosphingolipid, colon cancer (CRC) sphingolipidomics, sphingosine-1-phosphate, lactosylceramide

## Abstract

The development and progression of colorectal cancer (CRC), a major cause of cancer-related death in the western world, is accompanied with alterations of sphingolipid (SL) composition in colon tumors. A number of enzymes involved in the SL metabolism have been found to be deregulated in human colon tumors, in experimental rodent studies, and in human colon cancer cells in vitro. Therefore, the enzymatic pathways that modulate SL levels have received a significant attention, due to their possible contribution to CRC development, or as potential therapeutic targets. Many of these enzymes are associated with an increased sphingosine-1-phosphate/ceramide ratio, which is in turn linked with increased colon cancer cell survival, proliferation and cancer progression. Nevertheless, more attention should also be paid to the more complex SLs, including specific glycosphingolipids, such as lactosylceramides, which can be also deregulated during CRC development. In this review, we focus on the potential roles of individual SLs/SL metabolism enzymes in colon cancer, as well as on the pros and cons of employing the current in vitro models of colon cancer cells for lipidomic studies investigating the SL metabolism in CRC.

## 1. Introduction

The development and progression of colorectal cancer (CRC), which is a major cause of cancer-related deaths in the western world, is accompanied with substantial changes of cellular lipidome, including alterations of fatty acid, phospholipid, and sphingolipid (SL) composition in tumor tissue. Levels of numerous lipid species have been found to be significantly altered in colon cancer tissue (as compared with normal colon mucosa) [1,2]. This has initiated an intensive search for the mechanisms responsible for these alterations, focusing primarily on the changes in expression/activities of lipid metabolism enzymes, as well as studies aiming to decipher the structural and signaling functions of the individually altered lipids [3,4]. Uncovering the mechanisms responsible for lipid alterations during colon cancer development may bring important novel information about the process of colon cell transformation, as well as to provide novel targets for the prevention and/or treatment of this disease. Here, descriptive lipidomic studies may also help to identify potential lipid markers of distinct colon cancer stages. Although a number of studies have addressed the changes in CRC lipidome over the recent two decades, many aspects of this process are still not comprehensively covered in the current literature. In this review, we focus particularly on the potential roles of SLs in colon cancer, as well as on potential benefits or setbacks of the currently used in vitro models of colon cancer cells in lipidomic studies focusing on SL metabolism.

## 2. Currently Known Role(s) of SLs in Colon Cancer Cells—An Overview

SLs are bioactive molecules with a wide range of effects on key cellular processes, including cell growth, proliferation, and programmed cell death, as well as on the formation of membrane microdomains, endosomes, or secretion of exosomes [4,5]. The perturbations of SL metabolism, which include alterations of the responsible enzymatic pathways, may contribute to colon cancer progression and modulate responses of colon tumors to chemotherapy. Altered SL levels in colon cancer cells have been observed not only in vitro but also in animal experiments, as well as in some clinical studies [3,6,7]. Although some of the effects of SLs appear to be cell-specific, generally, increased intracellular levels of ceramides (Cer), sphingosines (Sph), and also dihydroceramides (dhCer) are mostly connected with the induction of cell cycle arrest and/or cell death, whereas the elevated levels of sphingosine-1-phosphate (S1P), ceramide-1-phosphate, glucosylceramides (GlcCer), and lactosylceramides (LacCer) seem to be associated with increased cell survival, proliferation, cell adhesion, and with the promotion of cell migration and/or invasion, the events associated with cancer progression [4]. Nevertheless, the exact functional roles of the changes in cancer cell sphingolipidome, which have been observed also during the multi-step process of colon carcinogenesis, are still only partially known. Until now, the changes in S1P/Cer ratio remain the best-characterized outcome of the alterations of SL metabolism in colon tumors [7].

### 2.1. Ceramides and Their Altered Metabolism in Colon Cancer Cells

Cer are central molecules of SL metabolism. In colon cancer cells, both Cer and S1P content seem to play significant roles in the pathogenesis of CRC, and the increased S1P/Cer ratio is an important characteristic of this disease [4]. Ceramidases, which hydrolyze ceramide into Sph, are key enzymes of SL metabolism. They help to maintain the cellular homeostasis through regulation of the balance between pro-apoptotic and proliferation-associated molecules [8]. For example, an increase of Cer levels in colon cancer cells treated with ceramidase inhibitors, or with Cer analogs, induced activation of the apoptotic cascade; treatment with ceramidase inhibitor also inhibited tumor growth in nude mice [9]. In a similar manner, Cer and sphingoid bases have been shown to inhibit tumor growth and to induce apoptosis in colon cancer cell lines [10]. Importantly, the biological activities of Cer seem to depend not only on the length of their covalently bound fatty acid residues, but also on the ratio between specific SL metabolites, which may play a decisive role in apoptosis regulation [11,12]. While upregulation of Cer 16:0 contributes to the induction of apoptosis in human colon cancer cells, increased Cer 24:1 levels are, in contrast, associated with cell survival [13]. Certain Cer species (16:0 and 24:1), as well as modulations of hexosylCer (HexCer) 24:1 and sphingomyelin (SM) 24:1 levels, could thus play significant roles in colon cancer cell life/death decisions, for example in response to therapeutic agents in vitro [14], or during the switch of cellular response of CRC-derived cell lines from differentiation to apoptosis [15].

The acid ceramidase (ASAH1) inhibition has been shown to exert an anti-proliferative effect in colon adenocarcinoma cells [16]. Similar to that, neutral ceramidase (ASAH2) has been identified as an important regulator of cell survival in several colon cancer cell lines. Kono et al. [17] have demonstrated, using Asah2 null mice, that ASAH2, which is predominantly expressed in the large intestine, could be the most important enzyme for the regulation of bioactive SL levels in the gastrointestinal tract. Inhibition of ASAH2 leads to an increase of Cer, which is accompanied with activation of apoptosis and autophagy, and a decreased survival of colon cancer cells, whereas its inhibition exerts only minimal effects in non-tumor intestinal cells. Inhibition of ASAH2 has been also reported to cause the loss of β-catenin and inhibition of extracellular signal-regulated kinase (ERK) signaling, thus blocking two important signaling pathways involved in colon carcinogenesis [18]. In another recent study, serine-threonine protein kinase AKT has been reported to be a key target for the growth-suppressive functions of Cer. Inhibition of ASAH2 decreased active (phosphorylated) AKT pool and induced dephosphorylation of GSK3β, which was then followed by phosphorylation and degradation of β-catenin and suppression of proliferation of colon cancer cells, both in vitro and in vivo [19]. Importantly, the activity of another ceramidase enzyme, human alkaline ceramidase 2 (ACER2), which increases the levels of both Sph and S1P, while simultaneously decreasing the levels of Cer, has been shown to be involved in the p53–mediated DNA damage response, as well as in regulation of cell cycle arrest and cellular senescence in colon cancer cells [20]. Modulation of activities of various ceramidase enzymes, leading to increased S1P/Cer ratio, may thus open promising opportunities for CRC therapy.

The family of ceramide synthases (CERS), consisting of six isoforms, is involved in the regulation of Cer content through the de novo synthetic pathway. Their expression has been shown to significantly modulate chemosensitivity of colon cancer cells. A recent study has shown that cytostatics, such as oxaliplatin or 5-fluorouracil, increase expression of CERS5 in a p53-dependent manner in human colon carcinoma HCT-116 cells. Downregulation of CERS5 has been found to be associated with enhanced sensitivity of CRC cells to chemotherapy, inhibition of autophagy and mitochondrial respiration [21]. Importantly, high CERS5 expression has also been found to be associated with reduced colon cancer patient survival; this could be mediated by a transition from apoptotic to autophagic signaling, and contribution of CERS5 to colon cancer progression [22]. CERS6 also belongs to the downstream mediators of p53-dependent apoptotic pathway. It has been reported that folate stress (induced by the expression of a major folate metabolism enzyme, ALDH1L1, or by folate depletion) leads to CERS6-dependent increase of Cer levels in human colon carcinoma HCT-116 cells [23], and that CERS6 could also be a target of anti-folate chemotherapeutic drug methotrexate [24]. Both CERS5 and 6, as well as their product, Cer 16:0, have also been shown to regulate post-mitochondrial cell death [25]. In this context, it is perhaps interesting that we have previously observed that induction of apoptosis in differentiating colon cancer cells is associated with up-regulation of CERS5 (which contributes primarily to Cer 16:0 production) and down-regulation of CERS2 (which contributes to Cer 24:1 production), as well as with deregulation of expression of other SL metabolism enzymes, namely with induction of sphingomyelin phosphodiesterase 1 (SMPD1) and down-regulation of dihydroceramide desaturase 2 (DEGS2) [15].

Immunohistopathological and gene expression analyses (in particular determination of altered mRNA levels of major components of SL metabolism) might also be a suitable predictor of survival in colorectal cancer. Aziz et al. (2016) have identified a gene signature which might be a reliable prognostic marker in CRC survival; among the genes linked with tumor development such as NOTCH2 or BRCA1, this set also included significantly decreased CERS6 in patient groups with poor prognosis of survival [26]. Another study has reported CERS2, CERS4, CERS5, and CERS6 to be significantly dysregulated in CRC, using six independent colorectal cancer cohorts [27]. However, so far, the role of different CERS in colon cancer progression is far from being completely understood, because the expression of various CERS genes may also partly depend on the stage/progress of CRC and the CERS mRNA levels might also reflect feedback loops resulting from distortions of SL metabolism. The specific roles of individual CERS and their products (long-chain or very-long-chain Cer) in the regulation of cancer cell proliferation [28], as well as in apoptosis and pro-survival autophagy [22], certainly deserve more attention.

Finally, increased Cer levels in cells may also result from the activities of sphingomyelinases (SMases), which hydrolyze SM to form Cer. Neutral SMases (SMPD2-4) have been found to be reduced in intestinal epithelial cells during colon carcinogenesis, and they have been proposed to contribute to apoptosis, e.g., through p53–mediated down-regulation of SMPD4 expression [29]. During the early stages of colon carcinogenesis, the activity of alkaline SMase (gene product of ENPP7) is downregulated. The decreased activity of SMases has been observed both in patients with familial adenomatous polyposis and in sporadic colon cancer [30]; however, this effect is probably not directly associated with mutations of the adenomatous polyposis coli (APC) gene [31]. Moreover, Chen et al. (2014) have reported that colon tumor incidence is higher in alkaline SMase knockout mice than in wild type mice [32]. In this study, the lack of alkaline SMase has been also found to be associated with an increased tumor size, which was accompanied with decreased Cer and increased S1P levels, as well as with both enhanced levels and nuclear translocation of β-catenin [32].

### 2.2. Modulations of Sph and S1P Levels in Colon Cancer

Sph, which is synthesized from Cer via activities of ceramidases, is another important regulator of intracellular β-catenin levels. Human colon cancer cell lines SW480 and T84 treated with Sph have been reported to exhibit reduced β-catenin content, both in cytosol and in the nucleus, which has been found to be associated with inhibition of cell proliferation and induction of apoptosis [33]. Treatment of cells with Sph also downregulates cyclin-dependent kinase 4 expression and reduces phosphorylation of retinoblastoma protein, two principal cell cycle regulators [34]. Sph can be phosphorylated by sphingosine kinases (SPHK) 1 and 2 in enterocytes to form S1P, a pro-survival molecule acting via a family of mammalian G–protein-coupled receptors, or, through additional signaling pathways [35]. SPHK1 is a major enzyme responsible for synthesizing S1P, and it has been documented to behave like an oncogene in various experimental systems [36]; its product S1P inhibits apoptosis, promotes proliferation and angiogenesis, and induces inflammatory signaling through activation of the nuclear factor kappa B (NF-κB) and STAT3 pathways [37]. Liang et al. (2013) have shown that S1P is necessary for the production of interleukin-6, an NF-κB regulated cytokine [38]. The pro-inflammatory effect of SPHK1 upregulation, and consequent increased expression of S1P receptor (S1PR1) can be further enhanced by Sphk2 deletion [38]. The potential role of SPHK2 enzyme in colon cancer could be a complex one. For example, it has been reported that, in contrast to exogenous SPHK2 over-expression, which increases cell cycle arrest or cell death, serum deprivation of CRC cells increases expression/activity of SPHK2, which is in turn linked with increased cell survival/proliferation [39]. This seems to support a pro-survival role of SPHK2 in CRC cells.

SPHK1 has been documented to be involved in cancer development in the APC^Min^ mouse model of colon carcinogenesis [34]. SPHK1 is highly overexpressed in human colon tumors, as well as in azoxymethane-induced aberrant crypt foci and colon tumors in rodents, where an increased SPHK1 expression seems to correspond with the progression phase of tumor development [34,40,41,42]. SPHK1/S1P pathway has been also implicated in the activation of arachidonic acid metabolism via cyclooxygenase 2 (COX2) induction and increased prostaglandin E2 (PGE2) production. Depletion of SPHK1 by RNA interference has been reported to significantly reduce COX2 expression and PGE2 production, to inhibit tumor development, as well as to reduce cell proliferation and increase apoptosis in colon cell models [40,43]. Finally, SPHK1 has been reported to promote malignant progression in colon cancer, and high SPHK1 expression seems to correlate with advanced tumor stages [42]. Together, these data imply that Sph and, in particular, S1P production may significantly contribute to colon cancer development and that the enzymatic pathways responsible for their formation could be targeted by enzyme-specific therapeutic approaches.

S1P can be also dephosphorylated by S1P phosphatases (SGPP1,2) and phospholipid phosphatases (PLPP1-3), or it can be irreversibly degraded by S1P lyase (SGPL1), which is highly expressed in enterocytes, and which has been found to be downregulated in colon cancer [37]. S1P phosphatase activities were found to be reduced in colon cancer patients [44]. SGPL1 acts as a tumor suppressor under cellular stress conditions, such as induction of DNA damage, hypoxia or serum deprivation. For promoting apoptosis in these conditions, SGPL1 requires both p53 and p38 activities [45]. Furthermore, SGPL1 has been shown to regulate a STAT3-activated, miRNA-mediated cell transformation during the process of colon carcinogenesis [37]. Just recently, functional roles of SGPL1 in colitis-associated colon cancer and inflammation have also been observed in Sgpl1 knockout mouse models [46].

### 2.3. Glycosphingolipids in Colon Cancer

Glycosphingolipids (GSLs), which are derived from Cer via a series of glycosylation steps, have been shown to regulate the expression of several genes associated with carcinogenesis [47]. Thus, GSLs can be expected to play important roles also during colon cancer development. One of HexCer—GlcCer—is synthesized by UDP-glucose ceramide glycosyltransferase (UGCG) in the first step of GSLs synthesis. The enzymatic activity of UGCG can reduce the levels of Cer, thus contributing to cellular escape from the Cer-induced apoptosis, and its overexpression has been found to be associated with multidrug resistance both in colon cancer cells [48] and in other cancer types [49]. Multidrug resistance protein 1 (MDR1, ABCB1), which belongs to the superfamily of ABC transporters, is directly upregulated upon UGCG activation. Importantly, MDR1 is responsible not only for the efflux of toxic xenobiotics (including cytostatics) from the cells, but it possesses also flipase activity, which is responsible for the transfer of GlcCer from cytosol to the lumen of Golgi apparatus, where LacCer are produced [49]. Additionally, UGCG activity could also be linked to other colon cancer cell resistance mechanisms; recently, it has been shown that inhibition of UGCG sensitizes colon adenocarcinoma SW480 cells, bearing a missense p53 mutation, to doxorubicin [50]. Increased levels of GSLs and increased UGCG can also be linked with increased cell proliferation, which has been documented in other cancer types [51].

Numerous (patho)physiological stimuli have been shown to activate the next step in GSLs synthesis through induction of LacCer synthases (B4GALT5, B4GALT6), including growth factors and pro-inflammatory cytokines [52,53]. LacCer can also be generated through catabolism of gangliosides and other complex GSLs via sialidases [54]. Accumulation of LacCer has been shown to be associated with induction of cell proliferation, adhesion, migration, and angiogenesis—processes linked with cancer progression [53]. Just recently, B4GALT5 has been identified as a potential novel target for the diagnosis and therapy in human CRC—increased B4GALT5 mRNA/protein expression and enzymatic activity have been observed in colon tumors, as compared with the normal colon tissue [55]. In colon cancer cells, LacCer may also inhibit apoptosis via stimulation of Bcl-2 expression [54], and LacCer may increase the activity of NAD(P)H oxidase leading to a mild ROS production followed by activation of pro-survival pathways, including ERK1/2 and NF-κB signaling, and I-cell adhesion molecule (I-CAM) upregulation, which may then lead to an increased cell proliferation and modulations of cell adhesion, migration or angiogenesis [53]. Last but not least, LacCer may serve as important precursors for the synthesis of more complex GSLs, and in the light of the recent discoveries discussed above, LacCer might become a useful CRC-specific marker.

More complex GSLs have also been suggested to play distinct roles in cancer development [56], and altered production of complex GSLs may also serve as a marker or therapeutic target in cancer [57,58,59]. A thorough description of their multiple roles in mammalian physiology and pathology seems to be out of the scope of this article. Nevertheless, it is important to mention here, at least briefly, a potential role of glycosidases, especially ganglioside sialidase (neuraminidase3, NEU3), which specifically hydrolyzes gangliosides, in CRC. Human NEU3 is markedly up–regulated in various cancers and it has been shown to suppress apoptosis in cancer cells [60,61]. NEU3 mRNA has been found to be highly increased in human colon cancer, in particular in the epithelial elements of adenocarcinoma, as compared with adjacent non-tumor mucosa; therefore, NEU3 might contribute to accumulation of LacCer in colon cancer [54]. NEU3 overexpression in cancer cells in vitro, such as in colon cancer HT-29 cell line, has been tentatively linked to the EGFR-mediated signaling, LacCer accumulation, stimulation of cell growth, and inhibition of apoptosis [62].

The data summarized above strongly suggest that both SLs and their glycosylated derivatives could play important roles in colon malignancies and their development. Nevertheless, the complex tumor microenvironment and tumor heterogeneity, which is typical also for colon tumors [63], as well as the intricacies of SL metabolism, these all make the identification of universal gene candidates, driving changes of SL levels in tumor epithelial cells, a significant challenge. Here, regulation of SL homeostasis in specific cell subpopulations, in particular within tumor epithelial-like cells, should be thoroughly investigated, as it may provide important information useful for CRC therapy suggesting novel combinations of anti-cancer targets. Our current study documents specific alternations of SL metabolism in epithelial EpCAM+ cells isolated from colon tumor tissue, which only partly overlap with gene expression changes detected in total colon tumor tissue samples [64]. Specific enzymes driving these changes are only beginning to be recognized, and the available data are often contradictory. Recently, increased mRNA levels of B4GALT5 gene have been found in colon tumor tissue of one patient cohort (*n* ≥ 4), while these were not observed in colon tumor biopsies of other patient cohorts (*n* ≥ 20), including our own data [26,64,65]. However, when the expression analysis of B4GALT5 gene was performed in EpCAM+ cells isolated from the same colon tumor samples, its mRNA levels were increased significantly. Importantly, the alterations in expression of the enzymes responsible for SL and GSL metabolism in EpCAM+ cells seem to be also accompanied with significant changes of specific classes of SLs, including SM, Sph, S1P, and LacCer [64]; this pattern is similar to the previous findings in total colon tumor samples discussed above.

**Figure 1:**
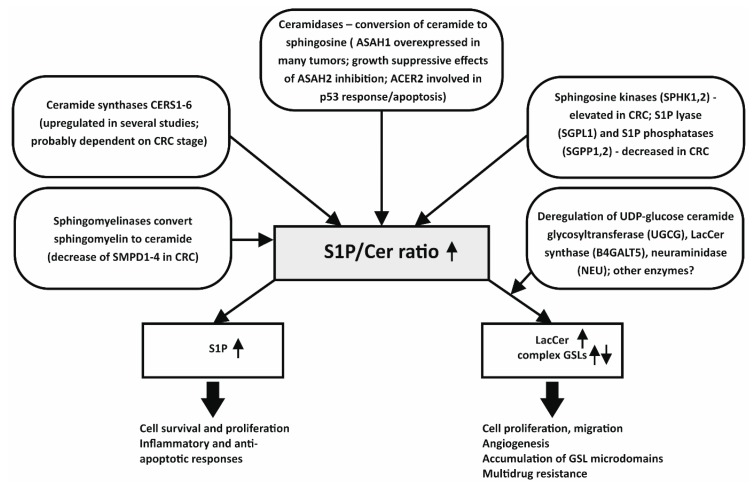
Deregulation of sphingolipid metabolism enzymes leads to increased S1P/Cer and LacCer/Cer ratios, associated with colon cancer progression. S1P, sphingosine-1-phosphate; Cer, ceramide; LacCer, lactosylceramide; GSLs, glycosphingolipids; CRC, colorectal cancer.

In conclusion, a number of genes/enzymes involved in SL metabolism have been found to be deregulated in human colon tumors, in experimental rodent studies or in human colon cancer cells in vitro. Many of them seem to be linked to an increased S1P/Cer ratio, which is, in turn, associated with increased colon cancer cell survival, proliferation, and cancer progression. However, the present data also suggest that more attention should be paid to the more complex SLs, including specific GSLs, such as LacCer, which appear to be significantly deregulated during CRC development. An overall summary of major SL metabolism pathways, deregulated during CRC progression, is provided in Figure 1.

## 3. Lipidomic Analyses of Human In Vitro Models of Colon Cancer Cells May Provide Important Insights into Deregulation of Bioactive Lipids, Including SLs

Although in vitro models of colon cancer cells have been intensively studied for complex changes in their transcriptomes upon various experimental conditions, and this information has helped to identify numerous therapeutic targets, there is a considerable lack of information about their lipidomic signatures, especially those regarding sphingolipidome. A comparison of global transcriptomic, lipidomic, and metabolomic data in well-characterized and stage-specific cancer cell models may point to novel CRC-associated processes, which might otherwise stay hidden in analysis of clinical samples due to tumor heterogeneity and inter-individual variability among CRC patients.

Characterization of changes in cellular lipidome during the adenoma–carcinoma transition could also be useful for discrimination of particular colon cancer stages, selection of specific colon cancer biomarkers, as well as for prediction of cellular responses to environmental factors, such as dietary lipids or therapeutic drugs. Our previous results have demonstrated the association of specific changes in lipid composition and metabolism, including various types of SLs, with modulation of proliferation, differentiation, and induction of cell death in colon cells, for example after treatment with dietary fatty acids and/or with endogenous regulators of tumor necrosis factor-αfamily of cytokines. These results have suggested that mutual interactions may exist between cellular lipidome and environmental factors, including dietary lipids, which may thus substantially alter cellular responses (apoptosis, differentiation) to treatment. Here, the cell transformation stage, as well as distinct differentiation capacities of colon cancer cells, seem to play important roles [14,15,66,67].

As summarized above, the cell lines derived from tumors at distinct stages of colon cancer development could potentially serve as useful models for the investigation of changes in individual lipids or lipid classes. The colon cancer-derived cell lines are, as colon cancer models, mostly characterized with regard to their genomic alterations, specific mutations or epigenetic changes [68]. Thus far, only a few studies have addressed alterations of lipidome in colon cancer cell lines [10,16,19,21,50]. Therefore, an important question is, whether colon cancer cell lines may also serve as suitable models for studies focusing on deregulation of SL metabolism, and whether the changes of sphingolipidome in CRC-derived colon cell lines, as compared with cell lines derived from normal colon tissue, may also reflect the alterations of sphingolipidome in colon tumors. Properly-designed lipidomic analyses comparing permanent epithelial colon cell lines established from normal, adenoma, adenocarcinoma and metastatic tissues, when coupled with suitable bioinformatics tools, may provide a new level of characterization of both differences and similarities among colon cancer cell models and also help to explain the differences in their responses to various agents.

Just recently, some specific SL metabolism alterations were observed in a series of colon cancer cell lines [69]. Here, surprisingly, the authors have found that SL content was very similar in NCM460 cells, derived from normal colon mucosa, and in SW620 cells, isolated originally from lymph node metastasis of colon carcinoma. This might perhaps reflect the changes associated with immortalization of NCM460 cells, or, a specific deregulation of SL metabolism in SW620 cells, as discussed below. Using a similar panel of colon cancer- and normal colon tissue-derived cell lines, we have, nevertheless, observed that phospholipid profiling enables us to discriminate colon cell lines according to their origin (distinct stage of colon cancer development). Moreover, we have also been able to identify some important similarities and discrepancies in phospholipid profiles between colon cancer cell lines and patient-derived cell samples [70].

**Figure 2:**
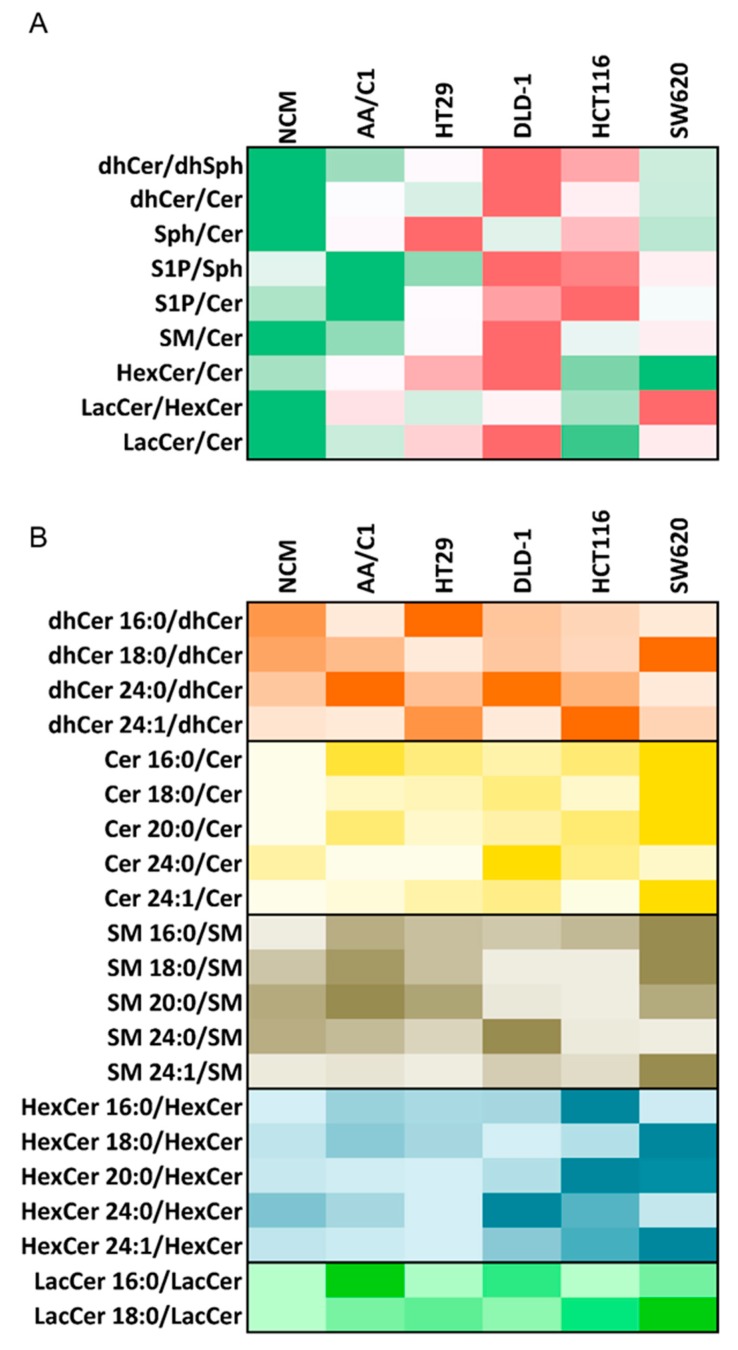
Fingerprint of sphingolipid ratios in cellular models of human colon cancer. Levels of individual sphingolipid (SL) species were analyzed using liquid chromatography–tandem mass spectrometry (LC-MS/MS) as in [15], using 1 × 10^6^ of cells representing different colon cellular models. Ratios specific for comparisons between SLs classes (**A**) and for individual SL species (**B**) were then calculated and plotted in the following manner: (**A**) red corresponds to the highest value of ratio (increasing color intensity reflects an increasing value of a given ratio), green to the lowest value of ratio (increasing color intensity reflects a decreasing value of a given ratio), (**B**) increasing color intensity reflects an increasing value of a given ratio.

Inspired by these observations, we report here original comparative, and so far unpublished, data on differences in sphingolipidome of colon cancer cell lines. We performed a highly detailed analysis of SL composition in six colon cell lines, using liquid chromatography-tandem mass spectrometry (LC-MS/MS) analysis, based on a method described before [15]. The analyzed cell lines included: NCM460 cells (derived from normal colon mucosa), AA/C1 cells (derived from adenoma), HT-29 cells (derived from non-invasive and differentiated carcinoma), DLD-1 and HCT-116 cell lines (derived from malignant carcinomas), and SW620 cells (derived from metastasis). Based on the results, we then first calculated product/substrate ratios based on total concentrations of major SLs (Figure 2A). Here, significant differences in relative SL content were found among individual cell lines. In colon carcinoma DLD-1 cells, we found the following changes in SL metabolism, as compared with normal colon mucosa NCM460 cells and colon adenoma AA/C1 cells:

1. An increased ratio for biosynthetic pathway (dhCer/dhSph), which was accompanied with an accumulation of dhCer (here shown as dhCer/Cer ratio) in carcinoma cells;
2. Increased S1P/Sph and S1P/Cer ratios indicating a higher relative content of S1P in carcinoma cells;
3. Increased levels of SM, as compared with Cer, in carcinoma cells;
4. Increased relative contents of HexCer and LacCer, as compared with Cer content in carcinoma cells.

All of these changes in SL metabolism in DLD-1 cells were in good accordance with the principal changes in sphingolipidome of human CRC patient samples that are discussed in Section 2. In another malignant carcinoma cell line, HCT-116 cells, we identified a similar pattern of changes in relative dhCer content and increased concentration of S1P, compared to Cer content; however, increase in GSL synthesis, which has just recently been reported in colon tumor samples [55], was not found in the HCT-116 cell line. The SW620 cells, isolated from distant metastatic tissue, showed a largely distinct pattern of SL ratios, being more similar to normal or to adenoma cells (Figure 2A). This seems to correspond to the previously published observation that, in terms of LacCer or GluCer levels, they are closer to NCM460 cells [69].

We then continued to evaluate the relative content of individual SL molecules (here expressed as ratios of individual species) within the respective SL classes (Figure 2B). Importantly, in DLD-1 cell line, an up-regulation of dhCer 24:0, Cer 24:0, SM 24:0, and HexCer 24:0 was observed. Importantly, these SL species are specifically linked with the induction of autophagy, cell survival and proliferation [11]. In contrast, only a low relative content of SL species with C24:0 structure was found in the metastatic SW620 cell line. Together with the observations of Peng et al. [69], this may suggest that this particular metastasis-derived cell line may not be representative of SL metabolism changes occurring in primary colon tumors and that the data obtained with this cell model should be interpreted with some caution.

Taken together, our data seem to suggest that at least some of established human colon cell line models could be potentially used in lipidomic studies, or as suitable in vitro models reflecting sphingolipidome alterations typical for colon tumors. Here, in particular, the DLD-1 cell line was identified as a promising model with an SL pattern similar to SL changes observed in colon cancer tissues.

## 4. Conclusions

CRC remains a principal cause of cancer–related death in the western world. The current evidence seems to support the hypothesis that alterations of SLs and their metabolizing enzymes could play important functions in CRC development and biology. However, the functional association of specific types of SLs (as well as other lipids) with CRC development is still only partly understood. Nevertheless, during recent years, significant progress has been made in this area, and deregulation of some specific sphingolipids has been found in a number of studies. In particular, Sph, Cer, and S1P have been identified as important bioactive lipids regulating key cellular functions, both in normal and in transformed colon cells. An increased S1P/Cer ratio, which may result from alterations of several pathways of SL metabolism, has been shown to contribute to CRC development through upregulation of cell survival and/or proliferation in tumor cells, while simultaneously promoting intestinal inflammation and reducing sensitivity of CRC tumors to chemotherapy.

Nevertheless, we are currently only starting to glimpse the whole scope of SL alterations in CRC. The changes in SL metabolism are highly dynamic and complex, and they may affect, and simultaneously be affected by, further steps in SL metabolism, including the formation of GSLs. Therefore, it seems important to evaluate the changes in SL species not individually, focusing on just a few isolated S1P or Cer species, but within the context of the whole sphingolipidome, ideally also in relation with the changes occurring simultaneously in triglyceride, cholesterol and phospholipid metabolism. Such complex analyses of colon cell lipidome may not only help us to identify new lipid-based CRC biomarkers but also to gain important insight into the roles of complex lipidome alterations in cellular mechanisms contributing to increased proliferation, survival, and resistance of CRC cells. One recent example is the identification of the up-regulation of LacCer synthesis as a potential marker for early CRC diagnostics.

The application of potential SL-based biomarker(s) could be hampered by an insufficient understanding of their biological significance. This is frequently complicated by the fact that most of the currently available data are derived from whole tumor tissue, which may contain numerous distinct cell types. Isolation of primary tumor cells, such as colon cancer epithelial cells, and their sphingolipidomic analyses may greatly aid to our knowledge about the roles of SLs in colon cancer cells. This can be further supported by a proper application of in vitro cell models derived from CRC cells, which may not always be straightforward. Nevertheless, upon a thorough evaluation of their SL metabolism, these cell lines, when sufficiently resembling primary tumor cells in terms of deregulation of SL metabolism (as it is demonstrated here, e.g., for DLD-1 cells), could be used as surrogate in vitro models of colon cancer cells in studies addressing, e.g., the functional roles of individual SL metabolism enzymes and pathways in CRC.

The present overview of deregulated genes and alterations of intracellular SL concentrations in colon cancer cells may help us to improve identification of major points of SL metabolism that could be targeted by the use of various antibodies, chemical inhibitors, shRNAs or a direct treatment with Cer and Cer analogs, e.g., encapsulated in nanoliposomes [71], or additional therapeutic approaches.

## Abbreviations

ACER2	alkaline ceramidase 2
AKT	AKT serine/threonine kinase
ALDH1L1	aldehyde dehydrogenase 1 family member L1
APC	adematous polyposis coli
ASAH1,2	acidic ceramidase 1 and 2
ASAH2	neutral ceramidase
B4GALT5, 6	beta-1,4-galactosyltransferase 5 and 6
C1P	Ceramide-1-phosphate
Cer	ceramides
CERS1-6	ceramide synthase 1-6
COX2	cyclooxygenase 2
CRC	colorectal cancer
DEGS	delta 4-desaturase, sphingolipid 2
dhCer	dihydroceramides
ENPP7	alkaline sphingomyelin phosphodiesterase
EPCAM	epithelial cell adhesion molecule
ERK	protein tyrosine kinase ERK
GlcCer	glucosylceramides
GSK3beta	glycogen synthase kinase 3 beta
GSLs	glycosphingolipids
HexCer	hexosylceramides
LacCer	lactosylceramides
LC–MS/MS	liquid chromatography–tandem mass spectrometry
MDR1 (ABCB1)	multidrug resistance protein 1
NEU3	neuraminidase/sialidase 3
NFκB	nuclear factor kappa B
PGE2	prostaglandin E2
PLPP1-3	phospholipid phosphatase 1-3
S1P	Sphingosine-1-phosphate
S1PR1	Sphingosine-1-phosphate receptor 1
SGPL1	Sphingosine-1-phosphate lyase 1
SGPP1,2	sphingosine-1-phosphate phosphatase 1 and 2
SL	sphingolipids
SM	sphingomyelins
SMase	sphingomyelinase
SMPD1-4	sphingomyelin phosphodiesterase 1-4
Sph	sphingosines
SPHK1,2	sphingosine kinase 1 and 2
STAT3	signal transducer and activator of transcription 3
UGCG	UDP-glucose ceramide glycosyltransferase
